# Phylogenetic constraints, conservatism, and convergence shape three‐dimensional variation in flower morphology of a tropical orchid radiation

**DOI:** 10.1111/tpj.70883

**Published:** 2026-05-05

**Authors:** Silvia Artuso, Alexander Gamisch, Yannick M. Staedler, Jürg Schönenberger, Hans Peter Comes

**Affiliations:** ^1^ Department of Environment and Biodiversity University of Salzburg A‐5020 Salzburg Austria; ^2^ Department of Botany and Biodiversity Research University of Vienna A‐1030 Vienna Austria

**Keywords:** 3D flower shapes, adaptive optima, *Bulbophyllum*, constraint, convergence, diversification, high‐resolution X‐ray computed tomography, phylogenetic comparative methods

## Abstract

Despite advances in phylogenetic comparative methods, challenges remain to distinguish between various macroevolutionary patterns of phenotypic variation (e.g., conservatism, convergence) and to infer their underlying proximate (genetic, developmental) or ultimate (selective versus neutral) causes. Furthermore, especially for complex morphological structures, little is known about the extent to which rates of trait and species diversification are coupled. To address these issues, we explored the modes and temporal dynamics of high‐dimensional (3D) flower shape evolution in Malagasy *Bulbophyllum* orchids (crown age: *c*. 12.7 Ma), representing the largest dataset of its kind in an angiosperm lineage (111 spp.). We identified three major flower shape types, which likely evolved under the influence of pollinator‐mediated selection around a “primary” (lineage‐wide) adaptive optimum over time. Moreover, we uncovered multiple instances of convergence and, in a recently derived subclade, a notable trend of shape “reversion” towards an earlier rather than more recent ancestral state. We conclude that the evolution of 3D flower shapes in orchids can be strongly influenced by phylogenetic constraint and conservatism while having no significant influence on species diversification. Hence, 3D flower shape (as potential pre‐zygotic reproductive isolation trait) may play less a role in explaining the diversification dynamics of orchids than generally assumed.

## Significance Statement

This study reveals that three‐dimensional flower shape in the Malagasy *Bulbophyllum*, a tropical orchid radiation, evolved under strong phylogenetic constraint and pollinator‐mediated selection rather than neutral drift. By integrating high‐resolution 3D morphometrics with phylogenetic comparative methods, we show that floral shape diversification proceeded primarily around a lineage‐wide adaptive optimum and was uncoupled from species diversification, providing new insight into how the evolution of complex floral traits influences orchid diversification.

## INTRODUCTION

Elucidating the patterns and processes of morphological (phenotypic) change and their relationship to species diversification are fundamental issues deeply rooted in evolutionary biology (e.g., Darwin, 1862; Haldane, 1932; Mayr, 1982; Stauffer, 1975; Thompson, 1917; Wright, 1956) and paleontology (Foote, 1994; Gould, 1989, 2002; Raup, 1985; Simpson, 1944). However, even with large and robust molecular phylogenies of extant species at hand, it often remains a challenge to distinguish among disparate macroevolutionary patterns of phenotypic variation (e.g., constraint, conservatism, convergence, reversion; Futuyma, 2010; Johnson et al., 2012; Porter & Crandall, 2003; West‐Eberhard, 2003) and to infer the underlying proximate (e.g., genetic, developmental) or ultimate (historical selective versus neutral) causes (*sensu* Mayr, 1982). For example, phylogenetic constraint (i.e., lineage‐specific limits on rates and/or direction of trait evolution due to shared phylogenetic history; Futuyma, 2010; McKitrick, 1993) or conservatism (i.e., retention of ancestral phenotypic traits; Ackerly, 2009) might be difficult to parse from repeated convergence, that is, the multiple/independent evolution of similar phenotypes in non‐sister lineages (e.g., Agrawal, 2017; Mahler et al., 2017; McLean et al., 2018). Similarly, trait conservatism/constraint may be driven by genetic‐developmental dependencies (e.g., Klingenberg, 2008, 2010) but could also reflect long‐term stabilizing selection (e.g., Davis et al., 2014; Felice et al., 2018; Hansen, 2014; Harmon et al., 2010; Lande, 1976). It also often remains unclear how a phenotypic trait is related to species diversification (e.g., Anderson et al., 2023; Donoghue & Sanderson, 2015; Helmstetter et al., 2023; Rabosky, 2014), and whether the trait *itself* has an influence or whether the association is with the *rate* of species diversification (Soltis et al., 2019).

Ancestral state reconstructions (ASRs) in combination with estimates of state‐dependent transition and speciation/extinction are still prevailing methods to model the timing, sequence, and convergence/reversion of discretely valued traits along molecular phylogenies and their impact on diversification (e.g., Gavrutenko et al., 2020; Ives & Garland, 2014; Pagel, 2004; Revell, 2013). However, for continuously valued traits, which contain higher statistical information (Paradis, 2014), recent advances in phylogenetic comparative methods (PCMs) can provide a more nuanced view on the mode and tempo of phenotypic evolution (e.g., Crouch & Ricklefs, 2019; Garamszegi, 2014). In this context, Brownian Motion (BM) has long been viewed as the dominant null hypothesis of neutral evolution (Ackerly, 2009; Felice et al., 2018; Felsenstein, 1985; Hansen, 1997, 2014). However, particularly for animals (e.g., reptiles, amphibians, fish), there is increasing evidence that the evolution of body size and complex biological structures (e.g., locomotor morphology, skull shape) rather follows an Ornstein–Uhlenbeck (OU) process (e.g., Citadini et al., 2018; Deepak et al., 2023; Harrington et al., 2018; Larouche et al., 2023; Oufiero & Gartner, 2014; Watanabe et al., 2019), which implies that, over time, ancestral phenotypic variance tends to drift towards one or multiple mean values viz. “optima” (Cressler et al., 2015; Hansen, 1997, 2014; O'Meara & Beaulieu, 2014). Also in flowering plants (angiosperms), there is increasing evidence that OU processes (with single or multiple different optima) have shaped the evolution of floral traits, including shapes and dimensions of organs (e.g., sepals, petals, corolla tube; Artuso et al., 2022; Hu et al., 2020; Joly et al., 2018; Kriebel et al., 2020) or even the three‐dimensional (3D) structure of entire flowers (Artuso et al., 2021; Dellinger et al., 2019; Reich et al., 2020), suggesting that pollinator‐mediated selection could play an important role in driving floral diversification (see also below).

Finally, we would expect from adaptive radiation theory (e.g., Glor, 2010; Schluter, 2000) that rates of species diversification and quantitative phenotypic evolution are coupled and initially increase, but then slowdown due to spatial/ecological niche filling and increasing competition (e.g., Moen & Morlon, 2014; Rabosky et al., 2013). In fact, an “early burst” (EB) of species accumulation is observed quite frequently, particularly in animals (Losos & Mahler, 2010; Rabosky & Lovette, 2008), but EB patterns of trait evolution appear to be rare (but see Agrawal et al., 2009; Clavel et al., 2019; Hertz et al., 2013; López‐Martínez et al., 2024). Likewise, a slowdown of species diversification coupled with decelerating phenotypic evolution has been observed in some animal and plant groups (Igea et al., 2017; Losos & Mahler, 2010; Price et al., 2014; Rabosky & Adams, 2012; Weir & Mursleen, 2013) but not in others (Désamoré et al., 2018; Folk et al., 2019; Rabosky et al., 2014; Slater et al., 2010; Testo & Sundue, 2018). Such decoupling between species and trait diversification might result from constrained phenotypic evolution, but this has rarely been noted explicitly (but see Artuso et al., 2021; Derryberry et al., 2011; Folk et al., 2019; Harmon et al., 2010). Clearly, further studies of the tempo and mode of both phenotypic and species diversification in particular lineages are needed before we will be able to identify universal principles underlying species radiations (see also Donoghue & Sanderson, 2015; Kennedy et al., 2022; Soltis et al., 2019).

In angiosperms, the flowers of orchids (Orchidaceae, *c*. 28 000 spp.; WCSP, 2025) are perhaps the most extensively studied morphological structures regarding both macro‐ and microevolutionary patterns and processes (e.g., Darwin, 1862; Givnish et al., 2015; Gravendeel & Dirks‐Mulder, 2015; Hu et al., 2020; Nilsson, 1992; Tremblay, 1992; Vereecken et al., 2010). The extraordinary floral diversity of Orchidaceae sometimes includes extreme divergence *and* convergence even among closely related taxa (e.g., Chase et al., 2009) and is generally attributed to pollinator‐mediated diversifying selection (e.g., Cozzolino & Widmer, 2005; Pérez‐Escobar et al., 2017; Rodríguez‐Otero et al., 2022; Schlüter & Schiestl, 2008) rather than non‐adaptive processes (but see Muchhala et al., 2014; Tremblay et al., 2005). The typical orchid flower has a complex, bilaterally symmetrical (zygomorphic) structure with three sepals, two lateral petals, and a well‐differentiated lip (“labellum”) (Dressler, 1993); the latter usually serves as the main attractive organ for pollinators (through colors, tactile cues, scent, etc.) and directs them to the fused androecium/gynoecium (“column”) for deposition and/or removal of pollinia (e.g., Rudall & Bateman, 2002). This tight integration (and partial multifunctionality) of organs could imply considerable constraints on orchid whole‐flower shape evolution. More specifically, over evolutionary time, strong stabilizing (*extrinsic*) selection for functionally effective trait combinations may have further promoted genetic‐developmental (*intrinsic*) constraints, as predicted for complex biological structures in general (Cheverud, 1982; Klingenberg, 2010; Monteiro & Nogueira, 2010; see also Gould, 1989, 2002). Consistent with this hypothesis, we found (Artuso et al., 2021) that whole‐flower shapes, as captured by high‐resolution X‐ray computed tomography (HRX‐CT) and high‐dimensional (3D) geometric morphometrics (3DGM), evolved in a constrained fashion, but uncoupled from species diversification, in a small‐sized clade (“*A*”; *c*. 38/50 spp.) of Malagasy orchids of the pantropical genus *Bulbophyllum* Thouars (Epidendroideae; *c*. 2200–2400 spp.; Pridgeon et al., 2014). However, it remains unclear to what extent these conclusions apply to the entire Malagasy *Bulbophyllum* lineage (*c*. 210 spp.; Sieder et al., 2009), whose tremendous floral morphological diversity is generally hypothesized to reflect the selective demands of different species belonging to their sole functional group of pollinators, that is, small Dipteran/*Drosophila*‐like flies (e.g., Artuso et al., 2021, 2022; Fischer et al., 2007; Hermans et al., 2021; Humeau et al., 2011; Pailler & Baider, 2020).

Based on molecular phylogenetic evidence (Gamisch et al., 2021; Gamisch & Comes, 2019), the radiation of *Bulbophyllum* began during the Mid‐to‐Late Miocene (*c*. 12.7 million years ago, Ma), followed by the formation of three main clades, termed *A* (*c*. 50 spp.), *B* (*c*. 126 spp.), and *D* (3 spp.), shortly afterwards (*c*. 12.7–11.6 Ma). Ancestral niche reconstructions further indicate that this monophyletic lineage likely originated in the sub‐humid rainforests of the “Central Highlands,” where most species are still found today, while only a few members of Clade *A* (sects. *Bifalcula* and *Calamaria*) later colonized the high‐rainfall coastal rainforest of the “Eastern Lowlands” and the seasonally dry “Sambirano” rainforest in the Northwest (Gamisch et al., 2021). The flowers of Malagasy *Bulbophyllum* are typically small (between 4 × 3 mm and 25 × 20 mm in size; Cribb & Hermans, 2009; Gamisch et al., 2014) and, characteristic for the entire genus, possess a specialized fly‐pollination system based on a mobile labellum attached to the base of the column (Artuso et al., 2022, and references therein). When triggered by the movement of an insect, the labellum closes and traps the pollinator, forcing it into contact with the column for pollinia removal. However, direct field observations of pollinators (small Dipteran flies) are available only for *B*. *cardiobulbum* in Madagascar (Chloropidae, *Arcuator* spp.; Hermans et al., 2021), and *B*. *variegatum* and *B*. *mascarenense* on La Réunion (e.g., *Platystomatidae* sp.: Humeau et al., 2011; *Mycetophilidae* sp.: Pailler & Baider, 2020).

In this article, we used the time‐calibrated phylogeny of Malagasy *Bulbophyllum* (179 spp.; Gamisch et al., 2021) as a framework to explore evolutionary variations in 3D flower shape among 111 species/specimens (including 38 spp. of Artuso et al., 2021), which represents the largest HRX‐CT/3DGM flower data set of an angiosperm lineage to date. Using 3DGM‐based ordination/clustering methods, we first identified major shape types (character states) and reconstructed their evolutionary history along the phylogeny to evaluate the relative importance of trait conservatism and convergence. In addition, we inferred state‐dependent transition and diversification rates. Finally, by adopting a PCM framework, we tested the fit of our high‐dimensional shape data to macroevolutionary models and compared rates of flower shape and species diversification through time. Overall, the results of the present study contribute to ongoing debates about the extent to which floral morphological diversity (or disparity) of Orchidaceae is influenced by phylogenetic constraint and convergence (e.g., Artuso et al., 2021; Chase et al., 2009; Schlüter & Schiestl, 2008), and whether 3D flower shape, as a potential trait of pre‐zygotic reproductive isolation (RI), has a role in species diversification (e.g., Soltis et al., 2019).

## RESULTS

### Identification and characterization of major flower shape types in Malagasy *Bulbophyllum*

**Figure 1:**
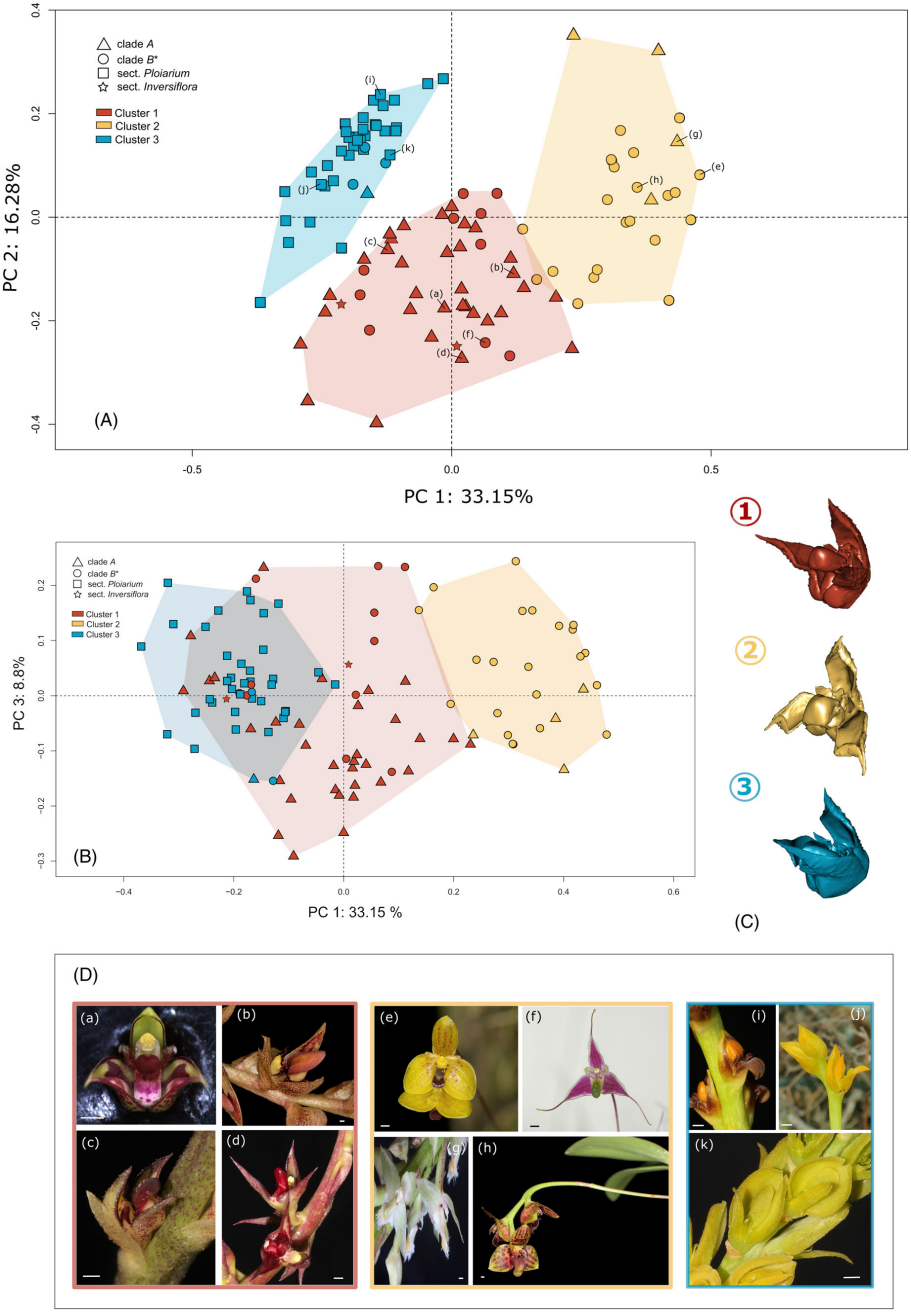
Identification of major flower shape types in Malagasy *Bulbophyllum*. Two‐dimensional principal component analysis (PCA) plots of flower shape variation among 111 Malagasy *Bulbophyllum* species for (A) PC1 versus PC2 and (B) PC1 versus PC3. Species are color coded according to their clustering into the three flower shape types (1, 2, and 3), while symbols represent clade/section affiliation, that is, Clade *A*; Clade *B* without species of sect. *Ploiarium* (Clade B*); sect. *Ploiarium*; and sect. *Inversiflora* (Clade *D*). Also shown are percentages of total variance explained by each PC. (C) Reconstructed 3D surface models of the three cluster averages. (D) Flower close‐ups of Malagasy *Bulbophyllum* species representing flower shape types 1 (framed red), 2 (yellow), and 3 (blue); (a) *B. bicoloratum* (*Calamaria*); (b) *B. variegatum* (*Alcystachis*); (c) *B. pusillum* (*Calamaria*); (d) *B. histrionicum* (*Calamaria*); (e) *B. analamazoatrae* (*Elasmotopus*); (f) *B. forsythianum* (*Lychenophylax*); (g) *B. imerinense* (*Kainochilus*); (h) *B. francoisii* (*Elasmotopus*); (i) *B. coccinatum*; (j) *B. auriflorum*; (k) *B. labatii* (all *Ploiarium*). Section names are given in parentheses. Bar, 1 mm. Photographs by Alexander Gamisch (a), Rudolf Hromniak (b, e, f, h), Peter Stütz (c, d, i, k, g), and Brigitte Ramandimbisoa (j; CC BY‐SA 4.0).

Of the 30 *k*‐means validity indices employed in nbclust, 13 and 12 suggested the partitioning of the 3D flower shape data of Malagasy *Bulbophyllum* (111 spp.) into two and three clusters (*K*), respectively (Table S3). After favoring *K* = 3 as being the more informative solution, we used anthropometry to assign the 111 flower shapes to three clusters, that is, type 1 (*N* = 45), type 2 (*N* = 26), and type 3 (*N* = 40). In the corresponding PCA plot (Figure 1A), the first component (PC1, explaining *c*. 33.15% of the total variance) widely separated samples of type 3 (dominated by species of sect. *Ploiarium* as part of Clade *B*) and type 2 (mainly the rest of Clade *B* spp.) into low (negative) and high (positive) scores, respectively; by contrast, samples of type 1 (mostly Clade *A* spp.) occupied an intermediate position (morphospace) along PC1 but tended to have lower scores along PC2 (16.3%). Notably, the plot of PC1 versus PC3 (8.8%; Figure 1B) revealed considerable overlap in the morphospace of type 3 and 1 (but not type 2) samples. Based on the average 3D flower shape surface models for each cluster (Figure 1C), types 1 and 3 both represent relatively closed flowers with a short/recessed labellum (compared to type 2). However, in type 1 (hereafter referred to as “closed”), the two lateral sepals are still separated by an angle of *c*. 45° (relative to the vertical flower axis), while in the “*Ploiarium*‐like” type 3, they are laterally joined and form a “boat‐like structure” (cf. Fischer et al., 2007); together with the dorsal (adaxial) sepal and short lateral petals, this results in a distinctly “bilabiate” flower (see also Hermans et al., 2021). By contrast, the “open” flowers of type 2 have widely separated (>90°) or even reflexed lateral sepals and a protruding labellum (see Figure 1D for representative species).

### Ancestral flower shape types and state‐dependent transition and diversification rates

**Figure 2:**
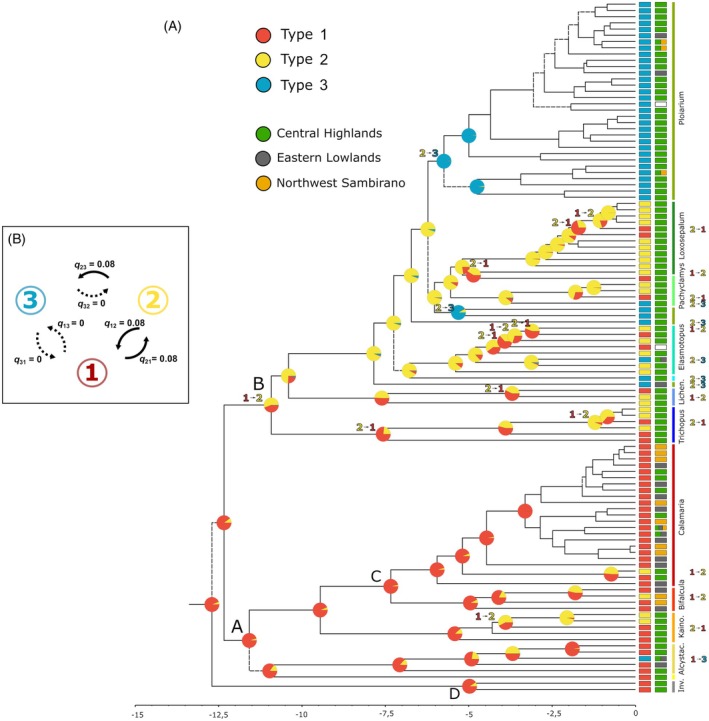
Evolution of 3D flower shape types in Malagasy *Bulbophyllum*. (A) Ancestral state reconstruction (ASR) of the three flower shape types (1–3) along the pruned maximum clade credibility (MCC) chronogram of Malagasy *Bulbophyllum* (111 spp.), using bayestraits. Pie charts at selected nodes indicate the relative probability (*P*) values of ancestral states. Numbers separated by small arrows indicate transitions between character states at relevant nodes. The tip species are color coded according to flower shape type (1–3) as well as phytogeographic zone/rainforest type (“Central Highland,” “Eastern Lowland,” “Northwest Sambirano”; see Gamisch et al., 2021). (B) Pairwise transition probabilities (*q*_12_, *q*_13_ … etc.) between flower shape types 1, 2, and 3 (circled numbers), as estimated by bayestraits. Dashed arrows indicate transition rates not significantly different from zero (see Table 1).

The bayestraits reconstruction of the three flower shape types along the MCC chronogram (111 spp.; Figure 2A) identified type 1 as the ancestral state of Malagasy *Bulbophyllum* with high probability (crown node *P* = 0.94; *c*. 12.7 Ma) and as a plesiomorphic condition retained by most species of Clade *A* (*P* = 0.95; *c*. 11.58 Ma; 38 spp.; see also Figure S1 for branch support). The transition to type 2 most likely coincided with the diversification of its sister lineage, Clade *B* (*P* = 0.74; *c*. 10.92 Ma; 73 spp.), in which type 3 subsequently evolved and was later fixed in the core *Ploiarium* subclade (*P* = 0.99; *c*. 5.75 Ma; 32 spp.), comprising most species of the non‐monophyletic sect. *Ploiarium*. By contrast, Clade *A* apparently experienced three independent transitions from type 1 to type 2, plus one to type 3, whereas the rest of Clade *B* (excluding the core *Ploiarium* subclade) probably underwent ten independent reversals from type 2 to type 1, plus seven independent transitions to type 3 (the latter involving nominal species of sects. *Elasmotopus*, *Pachyclamys*, and *Ploiarium*). However, considering the tip‐labeled phylogeny (Figure 2A), there was no obvious association of flower shape type (1–3) with phytogeographic zone/rainforest type (i.e., Central Highlands, *H*; Eastern Lowlands, *L*; and Northwest Sambirano, *S*).

Clearly, the above inferences about the number of independent transitions and reversals must be treated with some caution because ancestral states at several nodes appeared equivocal (Figure 2A; see also Gamisch et al., 2016). Nevertheless, these results qualitatively suggest relatively frequent state transitions, in particular between types 1 and 2 and from type 2 to 3, yet without providing evidence of type 3 reversals. In support of this, our bayestraits hypothesis testing (Table 1) strongly rejected models opposing transitions between types 1 and 2 (*q*_12_ or *q*_21_ = 0) or from type 2 to 3 (*q*_23_ = 0; all logBF >10), but none assuming zero transitions between types 1 and 3 (*q*_13_ or *q*_31_ = 0) or from type 3 to 2 (*q*_32_ = 0; all logBF <2). Overall, the rates of transition between types 1 to 2 (*q*_12_ and *q*_21_) and from type 2 to 3 (*q*_23_) were all estimated as 0.08 (see Figure 2B).

**Table 1:** Results of testing alternative models of transition (*q*) between the three flower shape types (1, 2 and 3) of Malagasy *Bulbophyllum*, using bayestraits

Model	LML	logBF
Unconstrained (full) model (M1)	−71.63	–
*q*_12_ = 0	−80.05	16.83
*q*_21_ = 0	−89.99	36.71
***q***_**13**_ **= 0**	**−71.59**	**−0.07**
***q***_**31**_ **= 0**	**−70.88**	**−1.48**
*q*_23_ = 0	−78.28	13.30
***q***_**32**_ **= 0**	**−71.70**	**0.15**

One unconstrained (full) six‐parameter model (M1), with free‐to‐vary transition rates, was compared to each of six constrained (simpler) models with one unidirectional (asymmetrical) transition set to zero. Each model was run over a sample of 100 trees from the Bayesian posterior distribution of the pruned maximum clade credibility (MCC) chronogram (111 spp.). The fit of each constrained model compared to M1 was assessed by calculating log marginal likelihood (LML) and log Bayes Factor (logBF) values. logBF = 2 (log marginal likelihood unconstrained, M1, model – log marginal likelihood constrained model). Note, the constrained model (null hypothesis, H_0_) was preferred when logBF < 2 (highlighted in bold), while logBF > 2 was considered positive evidence for the unconstrained (more complex) M1 model (strong, 6–10; very strong, >10; Kass & Raftery, 1995).

Our best supported MuSSE model (AIC = 631.14; Table 2) suggested a significant impact of flower shape type on rates of speciation (*λ*) but not extinction (*μ*), with higher and equal values of *λ* for types 2 and 3 (0.32) compared with type 1 (0.21). However, two zero‐extinction models with either lower and equal *λ* for types 1 and 2 (0.25) compared with type 3 (0.34) or equal *λ* for all types (0.28) performed almost equally well (ΔAIC = 1.19 and 1.20, respectively). In fact, all other models with zero extinction and at least two equal or free‐to‐vary speciation parameters had essentially an “equivalent” fit (ΔAIC <2; Table 2).

**Table 2:** Results from the MuSSE analysis in diversitree to test state‐dependent rates of speciation (*λ*) and extinction (*μ*)

Model	d.f.	*λ*_1_	*λ*_2_	*λ*_3_	*μ*_1_	*μ*_2_	*μ*_3_	Ln L	AIC	ΔAIC
Unconstrained (full) model (M1)	12	0.22	0.30	0.34	9.31E‐08	8.97E‐06	3.22E‐06	−309.10	642.20	7.83
*λ*_1_ = *λ*_2_ = *λ*_3_, *μ*_1_ = *μ*_2_ = *μ*_3_	3	0.28	0.28	0.28	1.41E‐06	1.41E‐06	1.41E‐06	−319.76	645.53	11.16
*λ*_1_ = *λ*_2_ = *λ*_3_	10	0.29	0.29	0.29	7.95E‐02	9.19E‐10	4.61E‐08	−310.44	640.88	6.52
*μ*_1_ = *μ*_2_ = *μ*_3_	10	0.22	0.30	0.34	1.59E‐07	1.59E‐07	1.59E‐07	−309.10	638.2	3.83
*λ*_1_ = 0	11	0	0.56	0.34	4.93E‐02	3.20E‐08	5.92E‐06	−318.79	659.59	25.22
*λ*_2_ = 0	11	0.38	0	0.34	1.2972E‐06	1.96E‐06	1.83E‐05	−318.11	658.22	23.85
*λ*_3_ = 0	11	0.23	0.63	0	5.5081E‐09	5.93E‐09	3.24E‐07	−332.38	686.76	52.39
*μ*_1_ = 0	11	0.22	0.30	0.34	0	7.72E‐09	3.05E‐08	−309.10	640.20	5.83
*μ*_2_ = 0	11	0.22	0.30	0.34	6.42E‐08	0	3.48E‐07	−309.10	640.20	5.83
*μ*_3_ = 0	11	0.22	0.30	0.34	1.51E‐07	6.56E‐07	0	−309.10	640.20	5.83
*μ*_1_ = *μ*_2_ = *μ*_3_ = 0	9	0.22	0.30	0.33	0	0	0	−309.10	636.20	1.83
*λ*_1_ = *λ*_2_ = 0, *λ*_3_	10	0	0	0.62	3.21E‐01	7.00E‐08	2.64E‐08	−365.20	750.40	116.04
*λ*_1_ = 0, *λ*_2_, *λ*_3_ = 0	10	0	0.84	0	4.18E‐01	5.14E‐07	1.02E‐01	−362.49	744.99	110.62
*λ*_1_, *λ*_2_ = *λ*_3_ = 0	10	0.58	0	0	1.11E‐06	6.31E‐09	1.65E‐01	−361.93	743.87	109.50
*μ*_1_ = *μ*_2_ = *μ*_3_ = 0, *λ*_1_ = *λ*_2_ = λ_3_	7	0.28	0.28	0.28	0	0	0	−310.78	635.57	1.20
*μ*_1_ = *μ*_2_ = *μ*_3_ = 0, *λ*_1_ = *λ*_2_	8	0.25	0.25	0.34	0	0	0	−309.78	635.55	1.19
*μ*_1_ = *μ*_2_ = *μ*_3_ = 0, *λ*_1_ = *λ*_3_	8	0.27	0.30	0.27	0	0	0	−310.70	637.39	3.02
***μ***_**1**_ **= *μ***_**2**_ **= *μ***_**3**_ **= 0, *λ***_**2**_ **= *λ***_**3**_	**8**	**0.22**	**0.32**	**0.32**	**0**	**0**	**0**	**−309.18**	**634.37**	**0**

An unconstrained (full) six‐parameter model, with free‐to‐vary state‐dependent rates of speciation (*λ*_1_, *λ*_2_, *λ*_3_) and extinction (*μ*_1_, *μ*_2_, *μ*_3_) was compared with several constrained models with parameters restricted to be either equal or zero (=0) (see Materials and Methods section). All models were fitted with a maximum likelihood (ML) nonlinear optimization based on the MCC chronogram of Malagasy *Bulbophyllum* (111 spp.). The fit of the models was assessed by computing Akaike information criterion (AIC) scores, considering models to be equivalent if ΔAIC < 2 (Burnham & Anderson, 2002). The preferred model (ΔAIC = 0) is highlighted in bold.

AIC, Akaike information criterion; Ln L, log‐likelihood.

### Testing for models of flower shape evolution

Our rpanda model testing (Table 3) clearly supported a (single‐optimum) OU model as best‐fitting the 3D flower shape data of Malagasy *Bulbophyllum* (lowest GIC value in 99% of the 100 randomized trees; BM: 1%; EB: 0%). However, the mean (±2 SD) estimate of *α* recovered under the OU model (0.52 ± 0.44) was small by common standards (*α* < 2; Beaulieu et al., 2012). The corresponding “phylogenetic half‐life” (mean *t*_1/2_ = 16.9 Myr; Table 3) exceeded (albeit not excessively) the total tree height (12.7 Myr), suggesting that the ancestral 3D flower shape of these tropical orchids evolved only slowly towards a long‐term mean value (*μ*) or primary adaptive optimum (e.g., Hansen, 2014; O'Meara & Beaulieu, 2014). Moreover, assuming an OU process, our PL‐MANOVA tests in mvmorph revealed a significant difference between the mean values of the clusters viz. morphospaces of the three flower shape types (Pillai's statistic = 1.852; *P* < 0.001), suggesting that they represent different adaptive optima.

**Table 3:** Evolutionary model fitting to the high‐dimensional (3D) flower shape data of 111 Malagasy *Bulbophyllum* species, using a penalized likelihood (PL) approach (Clavel et al., 2019), as implemented in rpanda

Model	Parameter (mean ± 2SD)	GIC (mean ± 2SD)	ΔGIC (2.5–97.5% range)	% trees preferred	Phylogenetic half‐life (*t*_1/2_) in Myr
BM	–	−104 585.77 ± 142	2.33–93.19	1	–
EB	*r* = 0	−104 583.77 ± 142	2.33–93.19	0	–
OU	*α* = 0.52 ± 0.44	−104 617.99 ± 146	0.01–0.36	99	16.9

Support for each model (i.e., BM, Brownian motion; EB, early burst; OU, single‐optimum Ornstein–Uhlenbeck) was evaluated over 100 randomized trees (see Materials and Methods section). The OU model is preferred in 99% of the trees, along with the lowest GIC value. For the OU model, the phylogenetic half‐life (*t*_1/2_) is reported (in million years, Myr). All models (BM, EB, OU) estimate *R*, the multivariate counterpart of *σ* ^2^, and the diffusion parameter of the univariate BM model, whereas the EB and OU models estimate an extra parameter (*r* and *σ*, respectively). Following Clavel et al. (2019), only the latter are shown here, whereas the Brownian parameters are given in the high‐dimensional *R* matrix (available upon request, along with correlational heatmaps).

GIC, Generalized Information Criterion; *r*, EB parameter of exponential rate decrease; SD, standard deviation; *α*, rate of adaptation parameter for OU; ΔGIC, difference in GIG value between the best model and the model being compared.

### Testing for a role of flower shape evolution in species diversification

**Figure 3:**
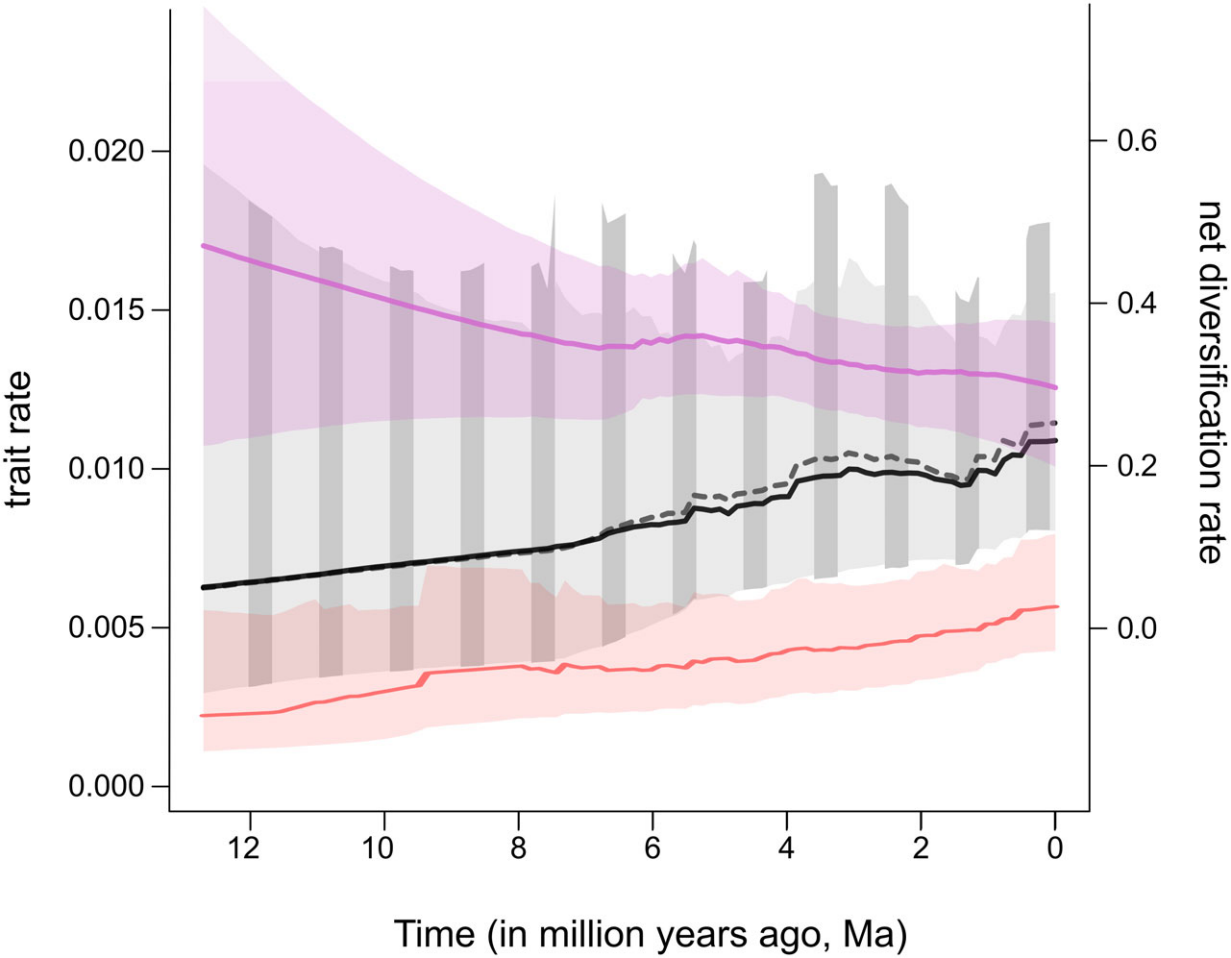
Relationship between rates of trait and species diversification in Malagasy *Bulbophyllum*. bamm‐derived rate‐through‐time (RTT) plots for flower shape evolution across 111 species of Malagasy *Bulbophyllum*, as computed for PC1 (black line) and PC2 (red line), along with their 95% confidence intervals (CIs; gray and light red, respectively). The dashed gray line represents the rate for PC1 after removing the core *Ploiarium* subclade from the analysis (with 95% CI shown as dark gray bars). Also shown is the bamm‐derived rate of net species diversification (*r*; dark violet line, with 95% CI in light violet) across the MCC chronogram of the entire Malagasy *Bulbophyllum* lineage (179 spp.; see Materials and Methods section).

We found no significant relationship between the net rate of species diversification (*r*) and flower shape type (1–3) or multidimensional shape space (PC1, PC2) across the 111‐spp. MCC chronogram (STRAPP analysis: all *P* > 0.98). Accordingly, flower shape *itself*, whether treated as a qualitative or quantitative trait, had no significant impact on the overall diversification of Malagasy *Bulbophyllum*. To further evaluate the relationship between the *rates* of flower shape evolution (in terms of PC1 or PC2) and *r* through time, respective RTT plots were generated in bamm (Figure 3). For PC1, the rate of flower shape evolution was initially low but then increased steadily (from *c*. 7.5 Ma), with some ups and downs, towards the present, irrespective of whether based on the 111‐spp. tree (black line) or after exclusion of the core *Ploiarium* subclade (dashed gray line; 32 spp.); the rate for PC2 (red line; 111 spp.) also tended to increase but remained low. By contrast, *r* (violet line), as re‐estimated from the 179‐spp. tree of Gamisch et al. (2021), decreased slowly over time. Hence, rates of flower shape evolution and net species diversification are uncoupled in Malagasy *Bulbophyllum*.

## DISCUSSION

### Phylogenetically constrained, stepwise, and reverted evolution of 3D flower shape types

Our GM and clustering analyses of 3D flower shape data for 111 out of *c*. 210 Malagasy *Bulbophyllum* species revealed three distinct clusters or shape *types*, corresponding to (1) “closed,” (2) “open,” and (3) “bilabiate” flowers, respectively (Figure 1A–D). Type 1 was found to represent the group's ancestral condition (Figure 2A), hence being present in the (most likely Asian) founder that colonized Madagascar during the Mid‐to‐Late Miocene (*c*. 12.7 Ma; Gamisch et al., 2021). Type 1 is still retained in most extant species of Clade *A* (crown age: *c*. 11.58 Ma). By contrast, the origin of type 2 dates to the crown node of Clade *B* (*c*. 10.52 Mya) and is still predominant there, except for type 3, which apparently originated in the early history of this clade and later became fixed in the core *Ploiarium* subclade (*c*. 5.7 Mya; Figure 2A). Overall, these results indicate that the evolution of flower shape types is constrained by phylogenetic history and essentially proceeded stepwise (from type 1 over 2 to 3) over relatively deep (Late Miocene) time scales (with some transitions/reversals; see below). Moreover, the greater similarity of species accessions characterized by type 3 to those of type 1 rather than type 2 (Figure 1A,B) implies a remarkable trend of “reversion” towards an earlier rather than a more recent ancestral state (e.g., Porter & Crandall, 2003; West‐Eberhard, 2003). However, it must be emphasized that type 3 does not represent an actual return to type 1, but more a *de novo* convergent form that *approximates* the more distant ancestral state (*sensu* Porter & Crandall, 2003). In turn, this resemblance already points to a lineage‐wide constrained trait evolution (see later).

### Phylogenetic conservatism outweighs trait convergence of flower shape types

According to our ASR analysis using bayestraits (Figure 2A), we inferred a relatively high number of transitions/reversals among the three flower shape types across the phylogeny (28 in total). These mainly occurred from type 1 to 2 in Clade *A*, or, more frequently, in the opposite direction in the “rest” of Clade *B* (i.e., without the core *Ploiarium* subclade), where type 2 also acted as ancestral state for transitions into type 3 (except for one from type 1 in Clade *A*). By contrast, reversals from type 3 to types 2 or 1 were not inferred and were also deemed improbable by the bayestraits model testing (Table 1; Figure 2B). Nevertheless, phylogenetic conservatism by far outweighs convergence of 3D flower shape types in Malagasy *Bulbophyllum*. This is perhaps not surprising for a complex, high‐dimensional “character,” in which function (sexual reproduction, pollinator attraction, etc.) depends on the fit or interaction among multiple organs (e.g., Artuso et al., 2022; Futuyma, 2010; West‐Eberhard, 2003). On the contrary, there is some need to explain the independent origins of type 2 (or rarely 3) in Clade *A* (relative to *B*) and of types 1 and 3 in the rest of Clade *B* (relative to *A* and the core *Ploiarium* subclade, respectively; see Figure 2A).

Such phenotypic convergences (or parallelisms) are often interpreted from two perspectives: shared genetic‐developmental (regulatory) pathways and similarities in selective environments (Futuyma, 2010; Higginson et al., 2012; McLean et al., 2018; Sanger et al., 2013; West‐Eberhard, 2003). As to the former explanation, we previously uncovered (Artuso et al., 2022) that the flower of *Bulbophyllum* comprises four modules (i.e., sepals, lateral petals, labellum, column) that largely reflect the differential expression of organ‐specific homoeotic (MADS‐box) genes (e.g., Hsu et al., 2015; Khojayori et al., 2024; Mondragón‐Palomino & Theißen, 2011; Wang et al., 2019). Hence, the repeated evolution of flower shape types in the Malagasy lineage could have been facilitated by this modular structure in combination with natural selection that can repurpose similar (or existing) regulatory pathways (e.g., Klingenberg, 2008; Pfennig & Pfennig, 2012). By contrast, the apparent irreversibility of type 3 (Figure 2B) could reflect conflicting selection pressures (e.g., from multiple pollinator species with opposite preferences for the same trait; Zhou et al., 2020) or too strong genetic‐developmental dependencies (Futuyma, 2010; Gould, 1989, 2002). On the contrary, there is little support yet for the (mutually non‐exclusive) hypothesis of shared selective environments. Thus, based on data from previous work (Gamisch et al., 2021), our tip‐labeled phylogeny (Figure 2A) indicates that the great majority of Malagasy *Bulbophyllum* species still share the group's ancestral Central Highland niche (*H*; except for most species of subclade “*C*” of Clade *A*). Hence, there is presently no evidence that macro‐ecological niche state viz. forest type (Gamisch et al., 2021) is associated with flower shape evolution in this tropical orchid radiation. Clearly, however, more detailed field and analytical research on fine‐scaled inter‐specific niche partitioning is needed to investigate whether shared selective environmental conditions (including pollinator abundances and composition) have driven the repeated evolution of the same flower shape type in this group.

### Which selective factors could have fostered the origins of shape types 2 versus 3?

According to our best‐supported MuSSE model (Table 2), both derived shape types (2 and 3) were associated with higher speciation rates when compared to the ancestral type (*λ*_2_ and *λ*_3_ = 0.32 versus *λ*_1_ = 0.22). However, the second‐ and third‐best models, assuming equal rates between shape 1 and 2 (*λ*_1_ and *λ*_2_ = 0.25 versus *λ*_3_ = 0.34) and for all three types (*λ* = 0.28), and likewise zero extinction, proved statistically equivalent (ΔAIC = 1.19 and 1.20, respectively). Hence, none of the derived shape types (2 and 3) are clearly identifiable as “key innovation” (*sensu* Donoghue & Sanderson, 2015) in having promoted higher species diversification relative to the ancestral form, nor did they trigger the colonization of Lowland (*L*) and Sambirano (*S*) forests (i.e., unlike CAM photosynthesis; see Gamisch et al., 2021). On the contrary, both derived shape types most likely evolved in the same ancestral habitat, namely the Central Highlands (*H*), as already “occupied” by type 1 (Figure 2A). It is feasible, therefore, that the divergence of these derived forms was primarily driven by competition‐ (or pollinator‐) mediated selection for floral traits involved in pollinator signaling, attraction, landing (etc.), thereby minimizing hybridization and/or increasing the potential for pollination isolation among broadly sympatric species (e.g., Armbruster & Muchhala, 2009; Grossenbacher & Stanton, 2014; reviewed in Moreira‐Hernández & Muchhala, 2019; see also below). In fact, this hypothesis of “reproductive character displacement” (RCD; *sensu* Pfennig & Pfennig, 2012) might also hold for the observed patterns of convergence (see above). One way to test this prediction is to investigate whether co‐occurring species of Malagasy *Bulbophyllum* are more different in 3D flower shape than expected due to chance at different (including allopatric) sites across the island (see also Cozzolino et al., 2022; Serrano‐Serrano et al., 2015).

### PCM‐based evidence for constrained 3D flower shape evolution

When treated as a continuous trait in a PCM framework, the evolution of 3D flower shapes in Malagasy *Bulbophyllum* best‐fitted an OU process (Table 3), implying constrained evolution towards a central mean value. This can be interpreted as evolution around an optimal shape but could also imply genetic‐developmental constraints (Artuso et al., 2021; Felice et al., 2018; Hansen, 2014). In addition, the corresponding estimates of *α* (0.52) and phylogenetic half‐life (*t*_1/2_ = 16.9 Myr) indicated a slow decay of ancestral effects and a low rate of adaptation towards a central mean value (Beaulieu et al., 2012; Cooper et al., 2016; Voje & Hansen, 2013). This is consistent with the phylogenetic conservatism of flower shape types deduced from the “discretely valued” ASR analyses (Figure 2A; see above). Finally, our PL‐MANOVA tests demonstrated that the three flower shape types themselves represent significantly different adaptive optima. Taken together, these results suggest that the predominant shape types of Clade *A* (1: “closed”), “rest” of Clade *B* (2: “open”), and the core *Ploiarium* subclade (3: “bilabiate”) moved towards different “secondary” optima, as likely fostered by pollinator‐driven selection/RCD (see above), but nonetheless evolved over time around a “primary” (lineage‐wide) shape optimum, culminating in the reversion of type 3 towards the more distant ancestral state of type 1 (Figure 1A,B).

The above OU processes, operating at both (sub)clade and lineage‐wide levels, could reflect shifts in the underlying genetic‐developmental architecture (e.g., Hansen, 2014). However, we consider this hypothesis to be less likely given the numerous instances of convergent evolution of flower shape types (see above). Instead, we suggest that the primary, lineage‐wide flower shape optimum of Malagasy *Bulbophyllum* species is mostly reflective of long‐term stabilizing selection (e.g., Hansen, 2014; O'Meara & Beaulieu, 2014), imposed by similar requirements of their single functional pollinator group (i.e., drosophilid flies; e.g., Hermans et al., 2021), for instance, in terms of foraging behavior or sensory ecology (e.g., Cresswell, 1998; Johnson, 2010; Richards, 1997). Concomitantly, the secondary optima (viz. flower shape types) may reflect adaptive responses to different guilds/species of fly pollinators. For example, despite our rudimentary knowledge of the fly‐pollinator composition and abundance of most Malagasy *Bulbophyllum* species (but see Artuso et al., 2021, 2022; Hermans et al., 2021; Humeau et al., 2011; Pailler & Baider, 2020), it is conceivable that their “closed” versus “bilabiate” flower types (1 versus 3) reflect selection for improved mechanical fit and/or avoidance of less efficient floral‐pollinator associations (e.g., Armbruster et al., 2009; Artuso et al., 2021, 2022; Fenster et al., 2004; Serrano‐Serrano et al., 2015). By contrast, the more open flowers of type 2 with a more protruding labellum might attract flies that have a stronger preference for complex shape outlines, as reported for other pollinator groups (e.g., Joly et al., 2018; Smith & Kriebel, 2018; see also Hu et al., 2022 for Asian *Bulbophyllum*). Regardless of these potential pollination mechanisms, it is important to emphasize that constrained (OU‐mediated) phenotypic evolution neither implies “stasis” nor excludes the possibility of selective processes at the species level (Hansen, 2014; O'Meara & Beaulieu, 2014). In fact, all three flower shape types encompass considerable inter‐specific morphological diversity viz. disparity (Figure 1), and flower shape (measured by linear or 2D geometric morphometrics) has experimentally been shown to be under strong pollinator‐mediated selection in other plant groups, including those sharing the same functional group of pollinators (e.g., Cuartas‐Domínguez & Medel, 2010; García et al., 2020; Gómez et al., 2014; Kopper et al., 2024); however, similar evidence for 3D flower shape is extremely rare due to the scarcity of relevant studies (but see Dellinger et al., 2023).

### Relationship between rates of flower shape evolution and species diversification

As previously shown only for Clade *A* (Artuso et al., 2021), the present results demonstrate that rates of flower shape evolution are not coupled to rates of net species diversification (*r*) even across the entire lineage of Malagasy *Bulbophyllum*. Specifically, we found no significant relationship of flower shape (whether treated as discrete or continuous (PC1, PC2) trait) with mean *r* (STRAPP: all *P* > 0.98) and observed a slowing‐down of *r* towards the present (Figure 3), while rates of flower shape variation continued to rise, especially for PC1 (*c*. ≤7.5 Ma; Figure 3). Notably, removal of the core *Ploiarium* subclade (fixed for the “bilabiate” shape type 3) from the RTT analysis had no (or even a slightly increasing) effect on the PC1 rate (Figure 3). This implies that shape diversity (in terms of PC1) mostly accumulated around the predominant “closed” versus “open” flower optima of Clade *A* and the rest of Clade *B* (types 1 versus 2), respectively. However, this long‐term increase of flower shape rate was likely further promoted by repeated state transitions, especially in the latter clade (see also Figures 1A,B and 2A).

Previous work (Gamisch et al., 2021) indicates that the slowing‐down of net species diversification (*r*) in Malagasy *Bulbophyllum* largely reflects decreased speciation (*λ*) rather than increased extinction (*μ*) and thus might be attributed to the filling of spatial/ecological niche space and increasing competition (e.g., Folk et al., 2019; Harmon et al., 2010; Moen & Morlon, 2014; Rabosky, 2009). However, such density‐dependent diversification not only results in a slowdown of *r* (see above) but should also favor morphological specialization (Futuyma & Moreno, 1988), accelerated phenotypic evolution (e.g., in RI traits) and/or increased partitioning of morphospace to permit species coexistence (e.g., Crouch & Ricklefs, 2019; FitzJohn, 2010; Monroe & Bokma, 2017). Hence, increasing competitive pressure (e.g., for pollinators) over time provides a possible explanation for the continued increase of flower shape rates (PC1 and to a lesser extent PC2) in the face of declining species diversity in Malagasy *Bulbophyllum*. In the future, it may be possible to test the validity of this hypothesis by examining how changes in species diversity over time affect the rate of 3D flower shape diversification under complex (e.g., multi‐peak OU) models of trait evolution.

## CONCLUSIONS

Our results show that the tremendous 3D flower shape diversity of Malagasy *Bulbophyllum* orchids comprises three major shape types, which likely evolved under the influence of pollinator‐mediated selection around a “primary” (lineage‐wide) OU optimum since the Mid‐to‐Late Miocene. We also identified multiple instances of convergence and a notable trend of shape “reversion” towards an earlier ancestral type in a recently derived subclade. Overall, the present findings, obtained from a single empirical example of an ancient (Mid‐to‐Late Miocene) tropical orchid lineage, serve to illustrate that 3D flower shape evolution in this group of organisms can be strongly influenced by phylogenetic conservatism and constraint while having no significant influence on species diversification. Accordingly, despite its potential importance as pre‐zygotic RI trait, 3D flower shape may play less a role in explaining orchid diversification dynamics than generally assumed. However, further investigations are needed to test whether constrained flower shape evolution and uncoupled trait‐diversification rates also hold for other lineages of Orchidaceae. Finally, it is worth emphasizing that our hypotheses about selective processes relate exclusively to the whole‐flower level and do not include other floral traits (e.g., size, color, microstructures, scent) that may have evolved along with shape for efficient pollination (e.g., Bogarín et al., 2018; Johnson & Steiner, 2000; Ramya et al., 2020; Wiśniewska et al., 2023).

## MATERIALS AND METHODS

### Study system and phylogeny

Our taxonomic sampling for flower shape analysis includes 111 of *c*. 210 species (53%) of the Malagasy *Bulbophyllum* lineage (Sieder et al., 2009), representing all major clades and sections, and with only a few species also occurring in the Mascarenes and East Africa (see Table S1). Phylogenetic data for these species were derived from a nuclear/plastid maximum clade credibility (MCC) chronogram (179 spp.; Gamisch et al., 2021), which dated the crown age of this monophyletic lineage to the Mid‐to‐Late Miocene (*c*. 12.7 Ma; see Introduction section). We trimmed this phylogeny to the 111 species matching our phenotypic data, using the R package Phytools v.0.6.99 (Revell, 2012; function *drop.tips*) (see MCC tree in Figure S1, with posterior probability (PP) estimates of branch support). For most of these 111 species, information is available regarding their rainforest types (Central Highlands (*H*); Eastern Lowland (*L*), and/or Northwest Sambirano (*S*); 108 spp.; Gamisch et al., 2016, 2021). As this phytogeographic (and climatic) element might be important for changes in flower shape (e.g., through associations with pollinator assemblages; e.g., Reyes et al., 2021), we tip‐labeled these rainforest types in the phylogeny of Figure 2A.

### HRX‐CT scanning and 3D flower modeling

For each of the 111 species, one open flower was harvested from either (1) ethanol‐preserved material deposited at the University Botanical Gardens of Salzburg (HBS) and Vienna (HBV) (107 spp.); or (2) herbarium specimens loaned from SZU, MO, and MNHN‐Paris (four spp.; Table S1). All plants were collected with permits issued by the Département des Eaux et Fôrets (Madagascar) and the Parc National de La Réunion, complying with relevant regulations. Information on the preparation of herbarium‐derived flowers and methods used for HRX‐CT scanning and 3DGM analysis can be found in Artuso et al. (2021). The HRX‐CT scanning of the 111 flower samples was performed following Staedler et al. (2013, 2018), and the 3D image stacks were created from the raw data using xmreconstructor v.8.1.6599 (Zeiss Microscopy, Jena, Germany). Using amira v.6.0 (Template Graphics Software Inc., San Diego, CA, USA), 52 landmarks (LMs) were added to each 3D‐flower model for the 3DGM shape analyses (see Figure S2 and Table S2 for LM placement and description, respectively). More information on LM placement accuracy, estimation of intra‐observer error, and a preliminary investigation of intra‐ versus inter‐specific shape variation in the sister species *B. bicoloratum*/*B. occultum* can be found in the Supplemental Information of Artuso et al. (2021).

### 3D geometric morphometric (3DGM) analyses

The following GM analyses were performed using the R package geomorph v.4.0.5 (Adams et al., 2020). After assessing the 3D coordinates of the 52 LMs for each species‐specific 3D flower model (see Artuso et al., 2021, for 10 spp. of Clade *A*), we subjected the full data set of the present study (111 spp.) to a Generalized Procrustes Analysis (GPA) superimposition (to separate shape from size, position, and orientation), removed potential effects of asymmetry (function *bilat.symmetry*), and used Phylogenetic Generalized Least Squares (PGLS) analysis to evaluate potential allometric effects of size on shape (function *procD.pgls*). Although the latter proved statistically significant (*P* = 0.006), size only explained 2.9% of the total shape variation, indicating a very weak and negligible effect by common standards (e.g., Al‐Shahrani et al., 2014; Camargo et al., 2019).

For the identification of major flower shape types, we first performed a principal component analysis (PCA) on the Procrustes‐fitted LM coordinates (geomorph function *gm.prcomp*). We then used nbclust v.3.0.1 (Charrad et al., 2014; function *NbClust*, method *K‐means*) on the first 14 principal components (PCs; >90% of the total variance) to determine the optimal number of clusters (*K*) in this multidimensional shape space based on 30 *k*‐means validity indices (see Table S3). Finally, we used anthropometry v.1.19 (Vinué, 2017) to apply Lloyd's *k*‐means algorithm directly to the Procrustes coordinates of the 3D shape data (function *LloydShapes*) to assign each species to one of the three clusters identified (i.e., shape types 1–3; see Results section). We also used two‐dimensional PCA plots to visualize the cluster membership of each species and reconstructed a hypothetical average 3D surface model for each shape type by warping the 3D surface file of a randomly chosen species (*B. senghasii*, Clade *A*) into their reconstructed average shapes (geomorph functions *mshape* and *warpRefMesh*).

### Ancestral state reconstruction (ASR) and state‐dependent transition/diversification analyses

For the ASR analysis of the three flower shape types (1–3) along the 111‐spp. MCC chronogram, we used the submodule “Multistate” in bayestraits v.3 (Pagel et al., 2004) together with a continuous‐time Markov model of discrete character evolution. Following Gamisch et al. (2021), the reversible‐jump (rj) Markov chain Monte Carlo (MCMC) analyses, accounting for model uncertainty, were run for 5 × 10^6^ generations, using a hyper prior with an exponential prior (mean seeded from a uniform distribution of [0, 100]) and a burn‐in of 5 × 10^4^ generations. Ancestral state probabilities (*P*), as derived from all visited models, were then plotted onto the chronogram for nodes of interest (i.e., crown, internal, and relevant near‐tip nodes; see Results section).

Next, we used the bayestraits hypothesis testing framework to identify the best‐fitting model for transition rates (*q*) of flower shape types along the MCC chronogram. Specifically, we compared an unconstrained (full) six‐parameter model (M1), with free‐to‐vary transition rates (i.e., *q*_12_, *q*_13_, *q*_21_, *q*_23_, *q*_31_, *q*_32_), to each of six constrained models with zero uni‐directional (i.e., asymmetrical) transitions for all pairwise combinations of states (i.e., *q*_12_ = 0; *q*_13_ = 0; … *q*_32_ = 0) (see Table 1). To incorporate phylogenetic uncertainty, each model was run over a sample of 100 trees from the Bayesian posterior distribution of the MCC chronogram (111 spp.). The average log marginal likelihood (LML) was calculated by stepping‐stone sampling (Xie et al., 2011). Model fit was assessed with log Bayes Factor (logBF) values. The constrained (simpler) model (null hypothesis, H_0_) was preferred when logBF <2, while logBF >2 was considered positive evidence for the unconstrained (more complex) model (strong, 6–10; very strong, >10; Kass & Raftery, 1995).

In addition, we constructed “multistate speciation and extinction” (MuSSE) models in diversitree v.0.9–18 (FitzJohn, 2012) to test if state‐dependent rates of speciation (*λ*) and extinction (*μ*) differ significantly from zero and between the three flower shape types (see Table 2). Again, we compared an unconstrained (full) six‐parameter model (M1), with free‐to‐vary state‐dependent rates of speciation (*λ*_1_, *λ*_2_, *λ*_3_) and extinction (*μ*_1_, *μ*_2_, *μ*_3_), to (1) a fully constrained model with equal rates of speciation (*λ*_1_ = *λ*_2_ = *λ*_3_) and extinction (*μ*_1_ = *μ*_2_ = *μ*_3_); (2) two models with either equal rates of speciation (*λ*_1_ = *λ*_2_ = *λ*_3_) or extinction (*μ*_1_ = *μ*_2_ = *μ*_3_), respectively; (3) six models with one parameter (rates of speciation or extinction) set to zero (i.e., *λ*_1_, *λ*_2_, *λ*_3_, *μ*_1_, *μ*_2_, or *μ*_3_ = 0); (4) one model with all extinction rates set to 0 (i.e., *μ*_1_ = *μ*_2_ = *μ*_3_ = 0); (5) three models with two speciation rates set to zero and one free to vary (e.g., *λ*_1_ = *λ*_2_ = 0, *λ*_3_); and (6) four models with extinction rates set to zero and different speciation rates tested to be equal (e.g., *μ*_1_ = *μ*_2_ = *μ*_3_ = 0; *λ*_1_ = *λ*_2_, *λ*_3_). All models were fitted with a maximum likelihood (ML) nonlinear optimization based on the MCC chronogram (111 spp.), with starting values for the parameters obtained (by default) from a state‐independent birth‐death (BD) model. The best‐fit model was determined based on the lowest Akaike information criterion (AIC) score, while models with a difference (ΔAIC) <2 were considered equivalent (Burnham & Anderson, 2002).

### Testing for models of flower shape evolution

For our PCM‐based analyses, we directly fitted three common models of continuous trait evolution (i.e., BM, single‐optimum OU, and EB) to the 3D shape data, using the penalized likelihood (PL) method of Clavel et al. (2019), as implemented in rpanda v.1.6 (Morlon et al., 2016; function *fit_t_pl*). Phylogenetic uncertainty was incorporated by running each analysis over a sample of 100 trees from the Bayesian posterior distribution of the 111‐spp. MCC chronogram. The best‐fit model was identified by the lowest Generalized Information Criterion (GIC) score (function *GIC*) and the highest percentage of trees preferring this model. Based on the OU‐derived “rate of adaptation” parameter *α*, we also calculated the “phylogenetic half‐life” as *t*_1/2_ = log_e_2/*α* (Hansen, 1997, 2014), where *t* is the time from the crown to the tip of the pruned MCC tree (here *c*. 12.7 million years, Myr; Gamisch et al., 2021). While *α* (ranging from zero to infinity) corresponds to the rate at which an ancestral phenotype evolves towards a long‐term mean value (*μ*) or primary adaptive optimum, *t*_1/2_ quantifies how much time is needed to lose half of the ancestral effect (Bartoszek et al., 2017; Hansen, 2014). Notably, the OU model becomes undistinguishable from the BM model when *α* is zero, or half‐lives are extremely long relative to the tree height (Beaulieu et al., 2012; Cooper et al., 2016).

As rpanda only allows testing a “single‐optimum” OU model, we additionally used the R package mvmorph v.1.1.7 (Clavel et al., 2015) to fit a multivariate PGLS model to our high‐dimensional 3D shape data by PL under the assumption of an OU process, using the three flower shape types as predictors (function *mvgls*) while accounting for measurement uncertainty of the data points (error parameter set to “true”). We then performed a multivariate analysis of variance (MANOVA) with Pillai's statistic (999 permutations) on the fitted model (function *manova.gls*) to test whether there is a significant difference in the mean values across the three types (viz. predictors), as would be expected if they represent different optima (J. Clavel, pers. comm.).

### Testing for a role of flower shape variation in species diversification

We first assessed whether flower shape *itself* is associated with net species diversification (*r* = *λ* − *μ*). To this aim, we used the STRAPP (Structured Rate Permutations on Phylogenies) procedure in bammtools v.2.1.10 (Rabosky & Huang, 2016) to test for a significant relationship of mean values of *r* at the tips of the 111‐spp. MMC chronogram with (1) the corresponding species scores for each principal component (PC1, PC2) of trait ordination, using Pearson's correlation coefficient, and (2) the three flower shape types, using a Kruskal–Wallis rank‐sum test (two‐tailed; 1000 permutations).

In addition, we used bamm v.2.5.0 (Rabosky, 2014) to compare evolutionary *rate*s through time (RTT) for net species diversification (*r*) and ordinated flower shape data (PC1 versus PC2), based on the full (179 spp.; Gamisch et al., 2021) and pruned (111 spp.) MCC chronograms, respectively. For each analysis, we performed one rjMCMC search of 10^7^ generations, sampling every 1000th step under the default prior assumption of a single expected rate shift. We used bammtools v.2.1.6 (Rabosky et al., 2014) for estimating priors (function *setBammpriors*), postanalysis, and visualization of RTTs (function *plotRateThroughTime*) after a 10% burn‐in. We also generated a separate RTT plot for PC1 of flower shape after excluding a derived subclade (nested within Clade *B*), as it was fixed for flower shape type 3 while containing most species of sect. *Ploiarium* (hereafter referred to as “core *Ploiarium* subclade”; see Results section). For the RTT analysis of net diversification (*r*), missing species could be accounted for by specifying a sampling fraction of 0.852 for the entire Malagasy lineage (i.e., 179/210 spp.; Gamisch et al., 2021).

## Author Contributions

SA, AG, and HPC designed the research. SA, AG, and YMS performed the research. SA and AG analyzed the data. SA and HPC wrote the manuscript. JS provided critical feedback and helped to interpret the results. All authors contributed to revisions and gave final approval for publication.

## CONFLICT OF INTEREST

The authors declare no conflict of interest.

## Supporting information


**Figure S1.** maximum clade credibility (MCC) chronogram of Malagasy *Bulbophyllum* (111 spp.), obtained by trimming the 179‐spp. MCC chronogram from Gamisch et al. (2021), using the R package Phytools v.0.6.99 (Revell, 2012; function *drop.tips*).
**Figue S2.** Landmarks (LMs 1–52; red dots) placed onto high‐resolution X‐ray computed tomography (HRX‐CT) scans of an exemplary flower of Malagasy *Bulbophyllum* (*B. francoisii* of sect. *Elasmotopus*).
**Table S1.** Sampling locations and voucher information for the 111 species of Malagasy *Bulbophyllum* included in this study.
**Table S2.** Description of the 52 landmarks (LMs 1–52), including 38 discrete and 14 semi‐LMs, used in the high‐dimensional geometric morphometric study of 3D flower shape variation among 111 species of Malagasy *Bulbophyllum*.
**Table S3.** Optimal number of clusters (*K*), as identified by each of the 30 *k*‐means validity indices in nbclust v.3.0.1 (Charrad et al., 2014; functions *K‐means* and *NbClust*), based on the PCA of 3D flower shape data of Malagasy *Bulbophyllum* (111 spp.), using the first 14 principal components (PCs; >90% of the total variance).

## Data Availability

Three‐dimensional image stacks of the raw flower scan data (TIFF format) as well as R scripts of the morphometric analyses (.zip files) are deposited on the public repository of the University of Vienna (https://phaidra.univie.ac.at/o:2170267).
