# Supplementary material for: Phylogenetic constraints, conservatism, and convergence shape three‐dimensional variation in flower morphology of a tropical orchid radiation
Source: Plant J. 2026 May 5;126(3):e70883. doi: 10.1111/tpj.70883 (PMC13143397; doi:10.1111/tpj.70883)
Supplement: Supplementary file 1 — Figure S1. maximum clade credibility (MCC) chronogram of Malagasy Bulbophyllum (111 spp.), obtained by trimming the 179‐spp. MCC chronogram from Gamisch et al. (2021), using the R package Phytools v.0.6.99 (Revell, 2012; function drop.tips). Figue S2. Landmarks (LMs 1–52; red dots) placed onto high‐resolution X‐ray computed tomography (HRX‐CT) scans of an exemplary flower of Malagasy Bulbophyllum (B. francoisii of sect. Elasmotopus). Table S1. Sampling locations and voucher information for the 111 species of Malagasy Bulbophyllum included in this study. Table S2. Description of the 52 landmarks (LMs 1–52), including 38 discrete and 14 semi‐LMs, used in the high‐dimensional geometric morphometric study of 3D flower shape variation among 111 species of Malagasy Bulbophyllum. Table S3. Optimal number of clusters (K), as identified by each of the 30 k‐means validity indices in nbclust v.3.0.1 (Charrad et al., 2014; functions K‐means and NbClust), based on the PCA of 3D flower shape data of Malagasy Bulbophyllum (111 spp.), using the first 14 principal components (PCs; >90% of the total variance). [file TPJ-126-0-s001.docx]

## Supporting Information

**Article title**:  **Phylogenetic constraints, conservatism, and convergence shape three-dimensional variation in flower morphology of a tropical orchid radiation**

**Authors**: Silvia Artuso, Alexander Gamisch, Yannick M. Staedler, Jürg Schönenberger and Hans Peter Comes

The following Supporting Information is available for this article.

**Fig. S1** Maximum clade credibility (MCC) chronogram of Malagasy *Bulbophyllum* (111 spp.), obtained by trimming the 179-spp. MCC chronogram from Gamisch *et al.* (2021), using the R package phytools v.0.6.99 (Revell, 2012; function *drop.tips*)

**Fig. S2** Landmarks (LMs 1–52; red dots) placed onto high-resolution X-ray computed tomography (HRX-CT) scans of an exemplary flower of Malagasy *Bulbophyllum* (*B. francoisii* of sect. *Elasmotopus*)

**Table S1** Sampling locations and voucher information for the 111 species of Malagasy *Bulbophyllum* included in this study

**Table S2** Description of the 52 landmarks (LMs 1–52), including 38 discrete and 14 semi-LMs, used in the high-dimensional geometric morphometric study of 3D flower shape variation among 111 species of Malagasy *Bulbophyllum*

**Table S3** Optimal number of clusters (*K*), as identified by each of the 30 *k*-means validity indices in NbClust v.3.0.1 (Charrad *et al*., 2014; functions *Kmeans* and *NbClust*), based on the PCA of 3D flower shape data of Malagasy *Bulbophyllum* (111 spp.), using the first 14 principal components (PCs; > 90% of the total variance)

**Figure S1.** Maximum clade credibility (MCC) chronogram of Malagasy *Bulbophyllum* (111 spp.), obtained by trimming the 179-spp. MCC chronogram from Gamisch *et al.* (2021), using the R package phytools v.0.6.99 (Revell, 2012; function *drop.tips*). Branches with posterior probability (PP) ≥ 0.85 are marked as solid lines and those with PP < 0.85 as dotted lines. Bars indicate 95% highest posterior density (HPD) intervals around mean node ages (in million years ago, Ma). Sectional affiliations of species are shown on the right side (coloured bars). Letters A–D highlight the crown nodes of the four major (sub)clades.

**
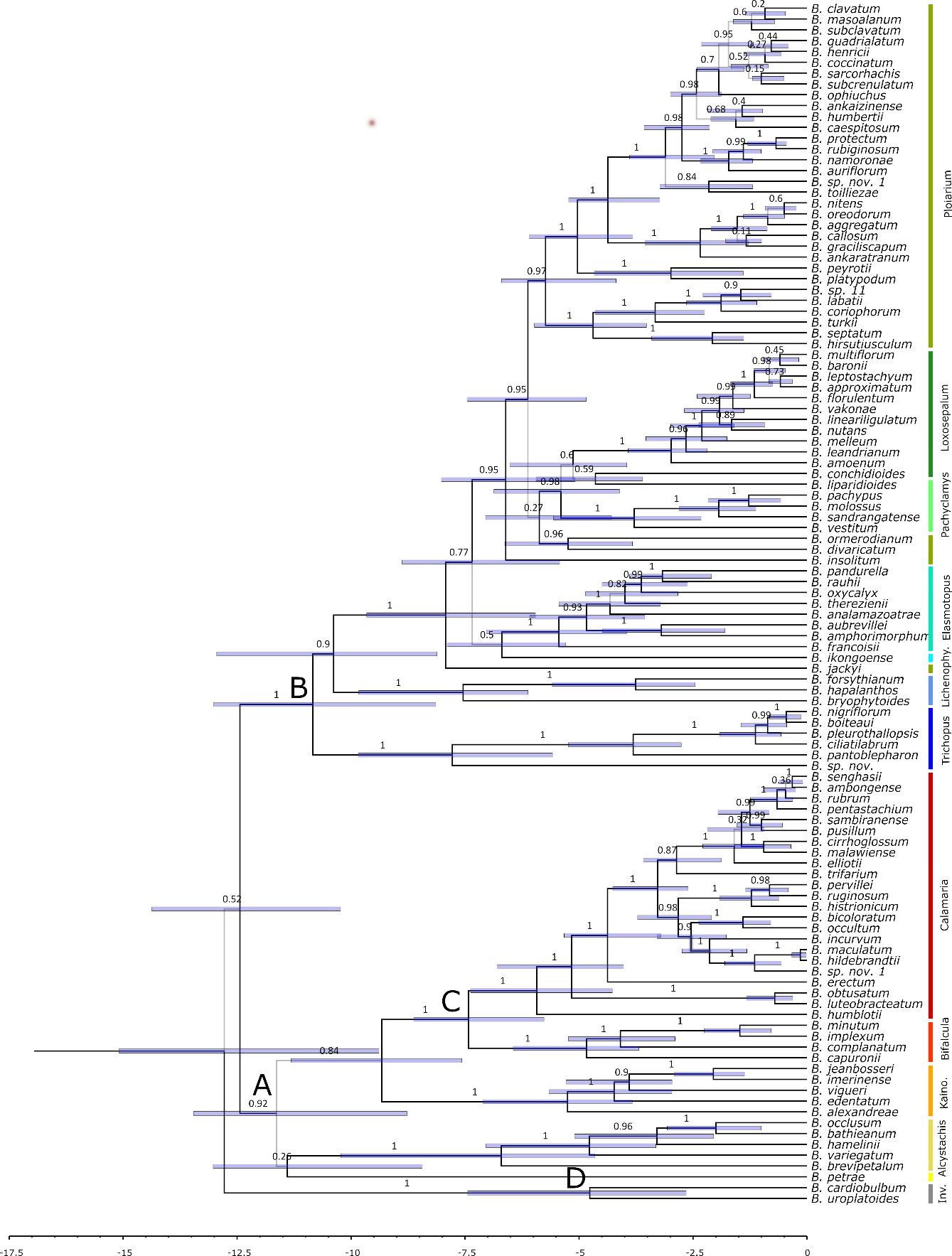
**

**Figure S2.** Landmarks (LMs 1–52; red dots) placed onto high-resolution X-ray computed tomography (HRX-CT) scans of an exemplary flower of Malagasy *Bulbophyllum* (*B. francoisii* of sect. *Elasmotopus*). (A) Front view of resupinate flower at anthesis, in more or less horizontal orientation. (B) Close-up of frontal view. (C) Flower seen from below (abaxial side). (D) Median (longitudinal) section. Refer to Table S2 for description of LMs.


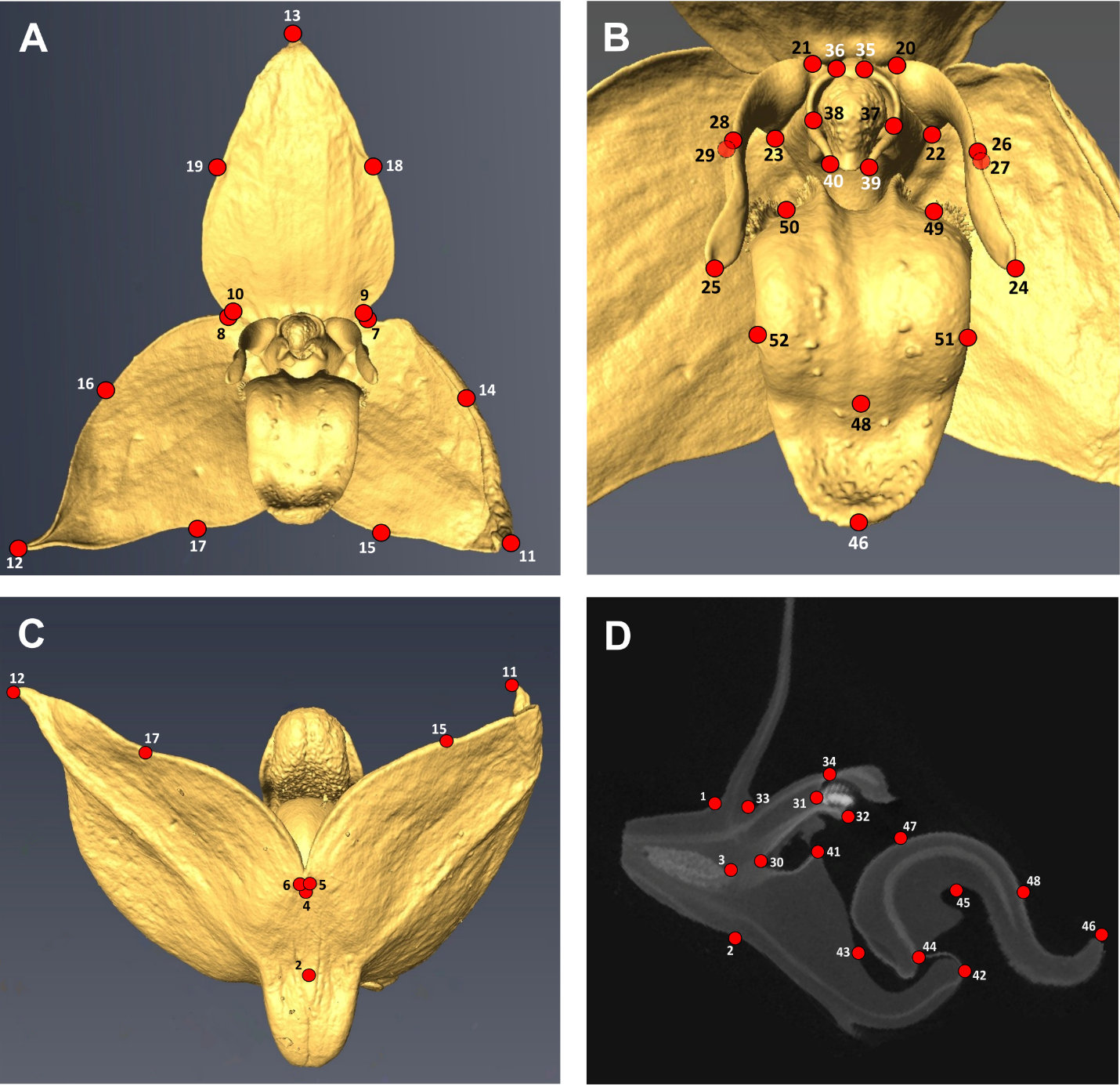


**Table S1** Sampling locations and voucher information for the 111 species of Malagasy *Bulbophyllum* included in this study. Further indicated are the two sample preparation methods used for high-resolution X-ray computed tomography (HRX-CT) scanning (IAA, in alcohol atmosphere; CPD, critical point drying) as well as the number (and codes) of landmarks (LMs) estimated per species (where applicable).

| Section/species | | Sample location ^1^ | Source (voucher) ^2^ | Collector ^3^ | Year of collection | Preparation method | Number (codes) of LMs estimated ^4^ |
| --- | --- | --- | --- | --- | --- | --- | --- |
| ***Alcistachys*** Schltr. | |  |  |  |  |  |  |
|  | *B. bathieanum* Schltr. | M | HBS/SZU (FS 5124) | GAF, JA | 2008 | IAA | – |
|  | *B. brevipetalum* H.Perrier | M | HBS/SZU (FS 2048) | GAF, JA | 2005 | IAA | – |
|  | *B. hamelinii* W.Watson | M | HBS/SZU (FS 3257) | GAF, WK, JA | 2006 | IAA | 3 (11, 12, 13) |
|  | *B. occlusum* Ridl. | M | HBS/SZU (FS 1501) | GAF, AS, WK, JA | 2004 | IAA | – |
|  | *B. variegatum* Thouars | RI | HBS/SZU (FS 799) | GAF, AS, JA | 2002 | IAA | 3 (11, 12, 13) |
|  |  |  |  |  |  |  |  |
| ***Bifalcula*** Schltr. | |  |  |  |  |  |  |
|  | *B. capuronii* Bosser | M | HBS/SZU (FS 1010) | GAF, AS, JA | 2002 | CPD | – |
|  | *B. complanatum* H.Perrier | M | HBS/SZU (FS 5756) | GAF, AS, JA | 2009 | IAA | – |
|  | *B. implexum* Jum. & H.Perrier | M | HBS/SZU (FS 6042) | GAF, AS, JA | 2009 | IAA | 1 (25) |
|  | *B. minutum* Thouars | M | HBS/SZU (FS 5306) | GAF, JA | 2008 | CPD | – |
|  |  |  |  |  |  |  |  |
| ***Calamaria*** Schltr. | |  |  |  |  |  |  |
|  | *B. ambongense* Schltr. | M | HBS/SZU (FS 6022) | GAF, AS, JA | 2009 | IAA | – |
|  | *B. bicoloratum* Schltr. | M | HBS/SZU (FS 5841) | GAF, AS, JA | 2009 | IAA | 1 (13) |
|  | *B. cirrhoglossum* H.Perrier | M | HBS/SZU (FS 4125) | AS, JA | 2007 | CPD | – |
|  | *B. elliotii* Rolfe | M | HBS/SZU (FS 863) | GAF, AS, JA | 2002 | IAA | – |
|  | *B. erectum* Thouars | M | HBS/SZU (FS 5383) | GAF, JA | 2009 | CPD | 3 (24, 11, 12) |
|  | *B. hildebrandtii* Rchb. f. | M | HBS/SZU (FS 5682) | GAF, AS, JA | 2009 | CPD | 1 (11) |
|  | *B. histrionicum* Rchb. f. ex G.A.Fisch. & P.J.Cribb | M | HBS/SZU (OR 1263_09) | GAF, AS, JA | 2009 | IAA | – |
|  | *B. humblotii* Rolfe ex Scott-Elliot | M | HBS/SZU (FS 1008) | GAF, AS, JA | 2002 | IAA | 12 (3, 20-23, 26-29, 32, 38, 40) |
|  | *B. incurvum* Thouars | RI | HBS/SZU (FS 1081) | Unknown | 2002 | CPD | – |
|  | *B. luteobracteatum* Jum. & H.Perrier | M | HBV (HBS/SZU (FS 5501) | GAF, JA | 2009 | IAA | – |
|  | *B. maculatum* Jum. & H.Perrier | M | SZU (HBS/SZU (FS 1133) | GAF, AS, WK, JA | 2004 | IAA | 9 (14, 15, 24, 26, 27, 46, 48, 51, 52) |
|  | *B. malawiense* B.Morris | MAL | HBS/SZU (OR 146_05) | Unknown | 2001 | IAA | – |
|  | *B. obtusatum* Schltr. | M | HBS/SZU (FS 5736) | GAF, AS, JA | 2009 | CPD | 3 (11, 14, 15) |
|  | *B. occultum* Thouars | M | HBS/SZU (FS 5028) | GAF, JA | 2008 | CPD | – |
|  | *B. pervillei* Rolfe | M | HBS/SZU (FS 818) | GAF, AS, JA | 2002 | CPD | 2 (12, 13) |
|  | *B. pusillum* Thouars | M | HBS/SZU (FS 2019) | GAF, JA | 2005 | CPD | 3 (24, 26, 27) |
|  | *B. pentastachium* (Pfitzer) Schltr. | M | HBS/SZU (FS 826) | GAF, AS, JA | 2002 | CPD | 1 (12) |
|  | *B. rubrum* Jum. & H.Perrier | M | MO (M3522007) | Unknown | Unknown | IAA | 4 (12, 3, 30, 41) |
|  | *B. ruginosum* H.Perrier | M | HBS/SZU (OR 1519_09) | GAF, AS, JA | 2009 | IAA | 1 (24) |
|  | *B. sambiranense* H.Perrier ex Hermans | M | HBS/SZU (FS 5718) | GAF, AS, JA | 2009 | CPD | – |
|  | *B. senghasii* G.A.Fisch. & Sieder | M | HBS/SZU (FS 3969) | OP | 2006 | CPD | – |
|  | *B. sp. nov. 1* | M | HBS/SZU (OR 1271_09) | GAF, JA | 2009 | IAA | – |
|  | *B. trifarium* Rolfe | M | HBS/SZU (OR 1576_09) | Unknown | Unknown | CPD | – |
|  |  |  |  |  |  |  |  |
| ***Elasmotopus*** Schltr. | |  |  |  |  |  |  |
|  | *B. amphorimorphum* H.Perrier | M | WU0063765 | AS, WK, CB, MP | 2008 | IAA | – |
|  | *B. analamazoatrae* Schltr. | M | 33099000 | Unknown |  | CPD | 11 (11, 12, 14, 15, 18, 19, 25, 34, 37, 39, 50) |
|  | *B. aubrevillei* Bosser |  | HBS/SZU (O00B155_12000) | AS, JA | 2000 | CPD | – |
|  | *B. francoisii* H. Perrier | M | HBS/SZU (FS 1419) | GAF, AS, KW, JA | 2004 | IAA | – |
|  | *B. oxycalyx* Schltr. | M | HBS/SZU (FS 1073) | GAF, AS, JA | 2002 | IAA | 2 (13, 40) |
|  | *B. pandurella* Schltr. | M | HBS/SZU (FS 6724) | Unknown |  | IAA | – |
|  | *B. rauhii* Toill.-Gen. & Bosser | M | HBS/SZU (FS 1326) | GAF, AS, KW, JA | 2004 | CPD | – |
|  | *B. therezienii* Bosser | M | HBS/SZU (FS 5556) | GAF, JA | 2009 | CPD | – |
|  |  |  |  |  |  |  |  |
| ***Inversiflora*** G.A.Fisch., Gamisch & P.J.Cribb. | |  |  |  |  |  |  |
|  | *B. cardiobulbum* Bosser | M | HBS/SZU (FS 6845) | Unknown | Unknown | IAA | – |
|  | *B. uroplatoides* [Hermans & G.A.Fischer](http://legacy.tropicos.org/Name/100368129?projectid=17) | M | HBS/SZU (FS 5133) | GAF, JA | 2008 | CPD | 2 (11, 13) |
|  |  |  |  |  |  |  |  |
| ***Kainochilus*** Schltr. | |  |  |  |  |  |  |
|  | *B. alexandrae* Schltr. | M | HBS/SZU (FS 2205) | GAF, AS, JA | 2005 | IAA | – |
|  | *B. edentatum* H.Perrier | M | HBS/SZU (FS 866) | GAF, AS, JA | 2002 | IAA | – |
|  | *B. imerinense* Schltr. | M | HBS/SZU (FS 2991) | AS, WK, JA | 2006 | IAA | – |
|  | *B. jeanbosseri* Gamisch & Hermans | M | HBS/SZU (FS 7342) | AS, AG | 2018 | IAA | 1 (3) |
|  | *B. viguieri* Schltr. | M | HBS/SZU (FS 2580) | GAF, JA | 2005 | IAA | 3 (11, 12, 13) |
|  |  |  |  |  |  |  |  |
| ***Loxosepalum*** Schltr. | |  |  |  |  |  |  |
|  | *B. amoenum* Bosser | M | HBS/SZU (OR226_03) | GAF, AS, JA | 2002 | CPD | 4 (11, 14, 15, 24) |
|  | *B. approximatum* Ridl. | M | HBS/SZU (WU0063555) | GAF, AS, JA | 2002 | IAA | – |
|  | *B. baronii* Ridl. | M | HBS/SZU (FS 1464) | GAF, AS, WK, JA | 2004 | CPD | – |
|  | *B. conchidioides* | M | HBS/SZU (FS 745) | GAF, AS, JA | 2002 | CPD | – |
|  | *B. florulentum* Schltr. | M | HBS/SZU (FS 1340) | GAF, AS, WK, JA | 2004 | IAA | 7 (6, 18, 19, 37/40) |
|  | *B. leandrianum* H.Perrier | M | HBS/SZU (FS 5149) | GAF, JA | 2008 | CPD | – |
|  | *B. leptostachyum* Schltr. | M | Unknown | Unknown |  | CPD | 3 (11, 14, 15­) |
|  | *B. lineariligulatum* Schltr | M | HBS/SZU (FS 1324) | GAF, AS, WK, JA | 2004 | CPD | – |
|  | *B. melleum* H.Perrier | M | HBS/SZU (FS 1651) | GAF, AS, WK, JA | 2004 | IAA | – |
|  | *B. multiflorum* Ridl. | M | HBS/SZU (FS 869) | GAF, AS, JA | 2002 | CPD | – |
|  | *B. nutans* (Thouars) Thouars | M | HBS/SZU (FS 2009) | GAF, JA | 2005 | IAA | 1 (11) |
|  | *B. vakonae* Hermans | M | HBS/SZU (FS 1911) | GAF, JA | 2005 | CPD | – |
|  |  |  |  |  |  |  |  |
| ***Lychenophylax*** Schltr | |  |  |  |  |  |  |
|  | *B. bryophytoides* G.A.Fischer & J. Andriantiana | M | HBS/SZU (FS 2546) | GAF, JA | 2005 | CPD | 3 (5, 11, 13) |
|  | *B. forsythianum* Kraenzl. | M | HBS/SZU (FS 2640) | GAF, JA | 2005 | IAA | 3 (11, 12, 13) |
|  | *B. hapalanthos* Garay | M | HBS/SZU (WU0063590) | GAF, AS, JA | 2002 | IAA | 3 (11, 12, 13) |
|  | *B. ikongoense* H.Perrier | M | HBS/SZU (FS 1575) | GAF, AS, WK, JA | 2004 | CPD | 1 (13) |
|  |  |  |  |  |  |  |  |
| ***Pachychlamys*** Schltr. | |  |  |  |  |  |  |
|  | *B. liparidioides* Schltr. | M | HBS/SZU (FS 706) | GAF, AS, JA | 2002 | IAA | – |
|  | *B. molossus* Rchb. f. | M | HBS/SZU (WU0063596) | GAF, AS, WK, JA | 2004 | IAA | 3 (13, 18, 19) |
|  | *B. pachypus* Schltr. | M | HBS/SZU (WU0063564) | GAF, AS, JA | 2002 | IAA | – |
|  | *B. sandrangatense* Bosser | M | HBS/SZU (FS 1661) | GAF, JA | 2004 | CPD | – |
|  | *B. vestitum* Bosser | M | HBS/SZU (FS 5182) | GAF, JA | 2008 | CPD | – |
|  |  |  |  |  |  |  |  |
| ***Ploiarium*** Schltr. | |  |  |  |  |  |  |
|  | *B. aggregatum* Bosser | M | HBS/SZU (FS 5142) | GAF, JA | 2008 | CPD | 1 (13) |
|  | *B. ankaizinense* (Jum. & H.Perrier) Schltr. | M | HBS/SZU (FS 1995) | GAF, JA | 2005 | CPD | 5 (30, 31, 32, 41, 51) |
|  | *B. ankaratranum* Schltr. | M | HBS/SZU (K72257000) | Unknown |  | CPD | 5 (11, 12, 13, 18, 19) |
|  | *B. auriflorum* H. Perrier | M | HBS/SZU (OR1225_09) | GAF, JA | 2009 | CPD | – |
|  | *B. caespitosum* Thouars | MU | MNHN-Paris (P0017296) | J. Bosser | 1976 | CPD | – |
|  | *B. callosum* Bosser | M | HBS/SZU (FS 625) | GAF, AS, JA | 2002 | CPD | – |
|  | *B. clavatum* Thouars | RI | MNHN-Paris (P02088221) | J. Bosser | 1972 | CPD | – |
|  | *B. coccinatum* H.Perrier | M | HBS/SZU (FS 5537) | GAF, JA | 2009 | CPD | 3 (21, 23, 25) |
|  | *B. coriophorum* Ridl. | M | HBS/SZU (OR257_03) | GAF, AS, JA | 2002 | IAA | 2 (37, 39) |
|  | *B. divaricatum* H.Perrier | M | HBS/SZU (FS 1463) | GAF, AS, WK, JA | 2004 | CPD | – |
|  | *B. graciliscapum* H.Perrier | M | HBS/SZU (FS 2002) | GAF, JA | 2005 | CPD | – |
|  | *B. henrici* Schltr. | M | HBS/SZU (FS 4725) | AS, WK, CB, MP | 2008 | CPD | – |
|  | *B. hirsutiusculum* H.Perrier | M | HBS/SZU (FS 1926) | GAF, JA | 2005 | CPD | – |
|  | *B. humbertii* Schltr. | M | HBS/SZU (FS 794) | GAF, AS, JA | 2002 | CPD | – |
|  | *B. insolitum* Bosser | M | HBS/SZU (FS 5492) | GAF, JA | 2009 | IAA | 8 (26/29, 37/40) |
|  | *B. jackyi* G.A.Fischer, Sieder & P.J. Cribb | M | HBS/SZU (WU0063585) | TA | 2006 | IAA | – |
|  | *B. labatii* Bosser | M | HBS/SZU (FS 737) | GAF, AS, JA | 2002 | IAA | – |
|  | *B. masoalanum* Schltr. | M | HBS/SZU (FS 2134) | GAF, JA | 2005 | CPD | – |
|  | *B. namoronae* Bosser | M | HBS/SZU (FS 6600) | Unknown |  | CPD | – |
|  | *B. nitens* Jum. & Perrier | M | HBS/SZU (FS 5146) | GAF, JA | 2008 | CPD | 1 (13) |
|  | *B. ophiuchus* Ridl. | M | HBS/SZU (FS 1480) | GAF, AS, WK, JA | 2004 | CPD | – |
|  | *B. oreodorum* Schltr. | M | HBS/SZU (WU0063622) | GAF, JA | 2005 | CPD | – |
|  | *B.* *ormerodianum* Hermans | M | HBS/SZU (FS 3149) | AS, WK, JA | 2006 | CPD | – |
|  | *B. peyrotii* Bosser | M | HBS/SZU (FS 2288) | GAF, JA | 2005 | IAA | – |
|  | *B. platypodum* H.Perrier | M | HBS/SZU (FS 4718) | AS, WK, CB, MP | 2008 | CPD | 1 (11) |
|  | *B. protectum* H.Perrier | M | HBS/SZU (FS 4207) | GAF, JA | 2007 | CPD | – |
|  | *B. quadrialatum* H.Perrier | M | HBS/SZU (FS 609) | Unknown |  | CPD | – |
|  | *B. rubiginosum* Schltr. | M | HBS/SZU (FS 800) | GAF, AS, JA | 2002 | IAA | – |
|  | *B. sarcorhachis* Schltr. | M | HBS/SZU (FS 2046) | GAF, JA | 2005 | CPD | – |
|  | *B. septatum* Schltr. | M | HBS/SZU (FS 3147) | AS, WK, JA | 2006 | IAA | – |
|  | *B. sp_11* | M | HBS/SZU (FS 624) | GAF, AS, JA | 2002 | IAA | – |
|  | *B. sp.* | M | HBS/SZU (FS 4418) | GAF, JA | 2007 | IAA | – |
|  | *B. subclavatum* Schltr. | M | HBS/SZU (FS 653) | GAF, AS, JA | 2002 | CPD | – |
|  | *B. subcrenulatum* Schltr. | M | HBS/SZU (FS 1669) | GAF, JA | 2004 | CPD | 1 (13) |
|  | *B. toilliezae* Bosser | M | HBS/SZU (K-22784.000) | Unknown |  | IAA | – |
|  | *B. turkii* Bosser & P.J.Cribb | M | HBS/SZU (FS 1595) | GAF, AS, WK, JA | 2004 | IAA | 1 (11) |
|  |  |  |  |  |  |  |  |
| ***Polyradices*** G.A.Fisch., Sieder & P.J.Gribb | |  |  |  |  |  |  |
|  | *B. petrae* G.A.Fisch., Sieder & P.J.Gribb | M | HBV (HBS/SZU (FS 2287) | GAF, AS, PJC | 2000 | IAA | – |
|  |  |  |  |  |  |  |  |
| ***Trichopus*** Schltr. | |  |  |  |  |  |  |
|  | *B. boiteaui* H.Perrier |  | HBS/SZU (FS 6601) | Unknown |  | IAA | – |
|  | *B. caniceps* Hermans, Sieder & Andriant. | M | HBS/SZU (FS 4073) | GAF, JA | 2007 | IAA | 3 (11, 14, 15) |
|  | *B. ciliatilabrum* H.Perrier | M | HBS/SZU (FS 604) | Unknown |  | CPD | – |
|  | *B. nigriflorum* H.Perrier | M | HBS/SZU (FS 1622) | GAF, AS, WK, JA | 2004 | CPD | – |
|  | *B. pantoblepharon* Schltr. | M | HBS/SZU (WU 0063594) | GAF, AS, JA | 2002 | CPD | 1 (40) |
|  | *B. pleurothallopsis* Schltr. | M | HBS/SZU (FS 1981) | GAF, JA | 2005 | CPD | 1 (3) |

^1^ Locality codes: M, Madagascar; MAL, Malawi, RI, Réunion Island (France), MU, Mauritius.

^2^ Source: HBS, Botanical Garden of Salzburg University; HBV, Botanical Garden of Vienna University; MO, Herbarium of the Missouri Botanical Garden; SZU, Herbarium of Salzburg University; MNHN-Paris: Muséum national d'Histoire naturelle.

^3^ Collector abbreviations: AG, Alexander Gamisch; AS, Anton Sieder; GAF, Gunter A. Fischer; JA, Jackie Andriantiana; OP, Olaf Pronk; PJC, P.J. Cribb; WK, W. Knirsch; CB, C. Berg; MP, M. Pinter; TA, T. Andriamihajarivo.

^4^ See Artuso *et al.* (2021) for details on how missing LMs were estimated, and Table S2 for identification of LM codes; “–“, all 52 LMs could be placed without adjustment.

**Table S2** Description of the 52 landmarks (LMs 1–52), including 38 discrete and 14 semi-LMs, used in the high-dimensional geometric morphometric study of 3D flower shape variation among 111 species of Malagasy *Bulbophyllum*. See Artuso *et al.* (2021) for details on landmark definition and placement, and Figure S2 for an exemplary illustration of landmark positions on a flower scan of Malagasy *B. francoisii.* The terms adaxial and abaxial are used here to indicate the position of the landmarks in the organs with respect to the inflorescence axis of the flower, at the resupinate stage.

| Code no. | Description |
| --- | --- |
| 1 | Adaxial side of the flower base |
| 2 | Abaxial side of the flower base |
| 3 | Starting point of the stylar canal in the middle section |
| 4 | Tip of the frontal conjunction between the right and left (lateral) sepals |
| 5 | Abaxial bulge of the right sepal base |
| 6 | Abaxial bulge of the left sepal base |
| 7 | Adaxial bulge of the right sepal base |
| 8 | Adaxial bulge of the left sepal base |
| 9 | Right bulge of the dorsal sepal base |
| 10 | Left bulge of the dorsal sepal base |
| 11 | Tip of the right sepal |
| 12 | Tip of the left sepal |
| 13 | Tip of the dorsal sepal |
| 14 | Semi-LM between LMs 7 and 11 |
| 15 | Semi-LM between LMs 5 and 11 |
| 16 | Semi-LM between LMs 8 and 12 |
| 17 | Semi-LM between LMs 6 and 12 |
| 18 | Semi-LM between LMs 9 and 13 |
| 19 | Semi-LM between LMs 10 and 13 |
| 20 | Adaxial side of the right petal base |
| 21 | Adaxial side of the left petal base |
| 22 | Abaxial side of the right petal base |
| 23 | Abaxial side of the left petal base |
| 24 | Tip of the right petal |
| 25 | Tip of the left petal |
| 26 | Semi-LM between LMs 20 and 24 |
| 27 | Semi-LM between LMs 22 and 24 |
| 28 | Semi-LM between LMs 21 and 25 |
| 29 | Semi-LM between LMs 23 and 25 |
| 30 | Base of the stigmatic cavity along the median section |
| 31 | Meeting point between the rostellum and the clinandrium |
| 32 | Distal point of the rostellum along the median section |
| 33 | Conjunction point between the column and the dorsal sepal, along the median section |
| 34 | Tip of the hinge between the column and the anther cap |
| 35 | Right side of the hinge between the column and the anther cap |
| 36 | Left side of the hinge between the column and the anther cap |
| 37 | Base of the right stelidium |
| 38 | Base of the left stelidium |
| 39 | Tip of the right stelidium |
| 40 | Tip of the left stelidium |
| 41 | Lower rim of the stigmatic cavity |
| 42 | Point where ligament is attached to the column |
| 43 | Semi-LM between LMs 41 and 42 |
| 44 | Point of ligament attachment to the labellum |
| 45 | Point of maximal curvature of the labellum on its abaxial side, along the median section |
| 46 | Distal end of the labellum |
| 47 | Point of maximal curvature of the labellum on its adaxial side, along the median section |
| 48 | Semi-LM between LMs 46 and 47 |
| 49 | Point of maximal curvature of the labellum’s right lateral lobe |
| 50 | Point of maximal curvature of the labellum’s left lateral lobe |
| 51 | Semi-LM between LMs 46 and 49 |
| 52 | Semi-LM between LMs 46 and 50 |

**Table S3** Optimal number of clusters (*K*), as identified by each of the 30 *k*-means validity indices in NbClust v.3.0.1 (Charrad *et al*., 2014; function *NbClust*, method *Kmeans*), based on the PCA of 3D flower shape data of Malagasy *Bulbophyllum* (111 spp.), using the first 14 components (> 90% of the total variance). Note, of the 30 indices tested, 13 and 12 favoured *K* = 2 and *K* = 3, respectively.

| Index | *K* | Index value |
| --- | --- | --- |
| KL | 3 | 4.0383 |
| CH | 2 | 42.3238 |
| Hartigan | 3 | 14.837 |
| CCC | 3 | 0.6855 |
| Scott | 3 | 201.427 |
| Marriot | 3 | 3.00E-04 |
| TrCovW | 3 | 0.429 |
| TraceW | 3 | 1.2374 |
| Friedman | 3 | 4.3707 |
| Rubin | 3 | -0.1633 |
| Cindex | 2 | 0.4587 |
| DB | 2 | 1.501 |
| Silhouette | 2 | 0.2763 |
| Duda | 2 | 1.3257 |
| PseudoT2 | 2 | -15.7234 |
| Beale | 2 | -2.2887 |
| Ratkowsky | 5 | 0.1549 |
| Ball | 3 | 2.406 |
| PtBiserial | 2 | 0.5957 |
| Gap | 2 | -0.5266 |
| Frey | 2 | 1.0827 |
| McClain | 2 | 0.5289 |
| Gamma | 2 | 0.712 |
| Gplus | 5 | 168.8727 |
| Tau | 2 | 1064.711 |
| Dunn | 3 | 0.2461 |
| Hubert | 0 | 0 |
| SDindex | 3 | 10.1313 |
| Dindex | 0 | 0 |
| SDbw | 5 | 0.3697 |
